# 组织因子在肺癌中的研究进展

**DOI:** 10.3779/j.issn.1009-3419.2010.09.13

**Published:** 2010-09-20

**Affiliations:** 430030 武汉，华中科技大学同济医学院附属同济医院普胸外科 Department of General Thoracic Surgery, Tongji Hospital, Tongji Medical College, Huazhong University of Science and Technology, Wuhan 430030, China

早在150多年前就有人提出恶性肿瘤患者容易出现高凝血状态，若血栓形成可能导致致命性并发症的发生。近年来恶性肿瘤和机体凝血系统的研究表明肿瘤不仅可以激活机体的凝血系统，而且后者参与肿瘤的恶性生物学行为。肺癌是目前世界范围内发病率和死亡率最高的肿瘤之一，组织因子作为机体凝血系统的重要始动因子，两者存在密切的关系。本文重点对肺癌中组织因子(tissue factor, TF)的表达以及在肺癌生长和转移中作用机制等加以综述。

## 1 TF的结构特征

TF的基因位于染色体1p21-1p22，总长度12.4 kb，含6个外显子所转录的mRNA为2.1 kb，最终翻译修饰的TF位于细胞表面相对分子量为47 kDa的一单链跨膜糖蛋白，属于二级细胞因子受体家族。它由263个氨基酸残基组成，分为含有可溶性的氨基末端的胞外区(丝氨酸1-谷氨酸219)、跨膜区(异亮氨酸220-亮氨酸242)和羧基末端的胞内区(组氨酸243-丝氨酸263)。胞外区有两个半胱氨酸对分别形成的二硫键组成稳定的二硫环，后者的羧基端以及该区的4个氨基酸残基在维持TF结构稳定性和促凝活性中起到了重要作用；跨膜区为疏水性的肽段，使TF锚定于细胞膜的磷脂胆碱中，而可溶性的TF分子活性较膜性TF大大降低，说明跨膜区可能有增强其分子活性作用；胞内区一个半胱氨酸245能与软、硬脂酸结合增加TF的跨膜稳定性，3个丝氨酸残基能被蛋白激酶(protein kinase C, PKC)激活与TF的功能密切相关^[1]^。

## 2 TF的凝血作用与类型

机体表达的TF在Ca^2+^的作用下，其胞外区FVII/VIIa受体能与血浆中的FVII结合，激活FVII形成TF-FVIIa复合物，后者再激活FX、FIX启动外源性凝血途径，产生凝血酶，使纤维素沉淀，血小板激活，最终血栓形成。根据TF是否与细胞膜结合分为膜结合性TF和可溶性TF，后者又根据是否与血液中的微粒结合分为具有促凝活性的微粒结合型TF(TF-microparticles, TF-MPs)和非微粒结合型TF，目前对后者的作用尚不了解。最近也有学者^[2, 3]^发现单核细胞表达产生全长型TF(full length TF, flTF)和选择剪切型TF(alternatively spliced TF, asTF)，后者因缺少正常TF基因的外显子5编码的跨膜区故为可溶性TF，其凝血活性较前者弱，但在新生血管的形成和肿瘤侵袭中可能起到了重要作用。

## 3 TF在肺癌中的表达

TF在某些组织细胞中如肺、心脏和脑等含量较多，但是正常情况下血液系统的单核细胞和血管内皮细胞等不表达TF，因此TF是唯一在正常人血浆中不存在的凝血因子。而在病理情况下，如组织损伤、炎症或肿瘤，细菌的脂多糖(lipopolysaccharide, LPS)、白介素-1(interleukin-1, IL-1)和肿瘤坏死因子(tumor necrosis factor, TNF)等可以促使血管内皮细胞和单核细胞或肿瘤细胞大量表达TF。它除存在于细胞表面外，在肿瘤患者血浆中也发现TF-MPs，可能是从肿瘤细胞表面脱落释放入血，从而启动凝血系统，导致血栓的形成。Giesen等^[4]^首次检测到TF-MPs具有凝血活性，并由体内模型中证明其在血管内血栓形成过程起到了关键作用。Zwicker等^[5]^发现在90例检测到TF-MPs的肿瘤患者静脉血栓栓塞症(venous thromboembolism, VTE)发生率为34.8%，而TF-MPs阴性的患者无一例出现VTE。

肺癌细胞表达TF^[6]^，而且其活性和肿瘤细胞的恶性转移倾向有关。小细胞肺癌(small cell lung cancer, SCLC)早期侵犯和转移能力强，NCI-H69细胞系(属于SCLC细胞系)表达的TF被证明与其强大的粘附和转移能力有关^[7]^，而周红等^[8]^研究发现高转移潜能肺癌系95D虽然表达的TF量低于低转移的LTEP-α-2肺癌细胞系，但活性远远高于后者。在患者的肺癌组织与血清中同样可以检测到TF的异常表达，并与肿瘤的类型、分期、预后相关。目前的研究认为SCLC表达的TF较非小细胞肺癌(non-small cell lung cancer, NSCLC)高^[9]^，腺癌较鳞癌高^[10]^，晚期肺癌较早期高^[11-13]^，出现远处转移或侵犯的患者高^[14]^，TF可作为判断肺癌患者预后的一个指标^[15]^。最近Rollin和Regina等^[11, 12]^检测NSCLC组织的flTF表达异常升高可能与*TP53*和*PTEN*等肿瘤基因的突变有关，但asTF升高与这些突变无关，因此asTF可能是NSCLC一种独立预测预后的标志物。Goldin-Lang^[15]^也发现肺腺癌组织中均有flTF和asTF的表达，但特别是asTF表达增强不仅预示了患者合并血栓的危险性增加而且提示患者的预后更差。

## 4 TF参与肺癌生长转移的信号途径

近年来对于TF和肿瘤关系的研究表明TF可以通过凝血和非凝血两条途径来促进肿瘤的生长和转移。其信号转导通路尚未完全明确，但是目前认为主要是蛋白酶活化受体(protease-activated receptors, PARs)家族介导信号的转导，PARs属于G蛋白偶联受体，分为PAR-1、PAR-2、PAR-3和PAR-4四种亚型，需要TF活化不同的凝血因子来激活。

凝血途径：TF与FVII形成活化的TF-FVIIa复合物激活肿瘤细胞表面PAR-2，之后的TF-FVIIa-FXa复合物可以激活PAR-1和PAR-2^[16]^，再通过p44/42 MAPK、p38 MAPK及JNK信号转导上调血管内皮生长因子(endothelial growth factor, VEGF)表达并抑制抗血管生成蛋白如凝血栓蛋白(thrombospondin-1, TSP-1)的表达^[17]^；其次FVIIa可使细胞内Ca^2+^浓度升高激活PKC，使胞内肌动蛋白结合蛋白-280(actin-binding p rotein 280, ABP-280)与TF胞浆尾区结合，上调MAPK信号活化局部粘附激酶(focal adhesion kinases, FAK)增加肿瘤细胞的粘附性和迁移性^[18]^。TF启动外源性凝血途径产生凝血酶(thrombin)，后者与PAR1、PAR3和PAR4结合通过MARK通路产生各种促肿瘤生长因子，如：VEGF、VEGFR、bFGF和MMP-2等^[19]^。

非凝血途径：TF受体胞内尾区的3个丝氨酸残基容易被胞内的PKC磷酸化，产生的信号上调各种促肿瘤生长因子表达^[20]^。

## 5 TF在肺癌进展中的作用

### 5.1 TF参与肿瘤性血栓形成

目前的研究^[21-24]^认为主要是血浆中的TF-MPs参与了肿瘤性血栓形成的过程。MPs来源于脂筏区的细胞质膜，富含TF、P-选择蛋白糖蛋白配体-1(P-selectin glycoprotein ligand-1, PSGL-1)和磷脂酰丝氨酸，单核细胞、血管内皮细胞、血小板以及肿瘤细胞表达的TF可以释放入血，成为TF-MPs，启动凝血系统。TF-MPs又可以通过自身PSGL-1和活化血小板表面P选择蛋白结合^[25]^，加速血栓形成的级联反应，形成大量微血栓。

游走的肿瘤细胞通过粘附素与血栓结合，同时肿瘤细胞表面的TF也可以使纤维蛋白沉积覆盖于肿瘤细胞自身，随着血栓的在血管中游走，肿瘤细胞随之转移^[16]^。Langer等^[26]^报道肺癌合并弥散性血管内凝血(disseminated intravascular coagulation, DIC)患者检测到血浆中TF-MPs有极强的促凝活性。肺癌合并VTE患者TF-MPs含量高于结肠癌等其它肿瘤患者，仅次于胰腺癌^[22]^。机体TF活性越强，不仅预示出现肿瘤合并血栓事件的危险性越高，而且可能加速肿瘤的血运转移，使肺癌患者的生存期缩短。Tesselaar等^[24]^认为腺癌患者合并VTE与TF-MPs活性升高密切相关，报道1例双下肢特发性深静脉血栓的患者，其TF-MPs活性异常升高，1个月后最终诊断为肺腺癌，故他们提出VTE患者若同时伴TF-MPs异常预示有患腺癌可能。但这一设想还需通过进一步的临床研究来证实。

### 5.2 TF参与新生血管的生成

TF在血管形成中扮演了重要的角色，胚胎期如果缺乏TF的表达会出现血管周皮细胞异常，卵黄囊血管发育缺陷，导致胚胎的早期死亡。不仅在生理情况下，病理条件下对肿瘤新生血管的形成也起到了关键的作用。VEGF是目前已知最强的直接作用于血管内皮促其增殖的因子并可增加血管的通透性，参与肿瘤新生血管的形成和生长转移^[27]^。没有血管的形成，肿瘤就无法生长。目前认为TF可以上调VEGF的表达同时抑制抗血管生成蛋白如凝血栓蛋白-1(thrombospondin-1, TSP-1)来促进肿瘤血管形成，其机制主要通过TF胞浆尾区的信号转导以及形成的TF-FVIIa复合物、凝血酶作用激活细胞表面的PARs胞内信号转导促VEGF基因表达^[28]^。

研究^[29, 30]^发现肺癌细胞表面均表达TF和VEGF，在肺癌组织中TF的表达水平与肿瘤血管密度、VEGF的表达量呈正相关，三者的表达量越高，肺癌的分期越晚，预后越差，出现远处转移的可能性越大。VEGF包括165、189、121和206个氨基酸的四种异构体，其中VEGF189主要功能与肺癌血管的新生和转移密切相关^[31]^。Regina等^[13]^使用RT-PCR检测64例NSCLC组织中TF、VEGF189和VEGF165 mRNA的水平，发现肺癌主要表达VEGF189，其mRNA在肺癌中的表达量是VEGF165的10倍，在*K*-*ras*突变的癌组织中VEGF189和TF表达均明显升高。

TF上调VEGF的同时，后者也可以正反馈促TF表达。Kim等^[32]^报道肿瘤组织中的内皮细胞的VEGF通过促进p38和Erk-1/2 MARPK活性来阻断可以抑制TF表达的PI3-K-Akt信号，使TF能够大量表达。

总之，TF诱导VEGF的表达，促进肿瘤原发和转移部位新生血管的形成，而肿瘤生长分泌大量的VEGF又可以使TF表达增强，导致微血栓的形成，肺部血供丰富，肿瘤细胞更容易远处转移。两者即以这种方式参与了肿瘤生长和凝血系统激活的恶性循环。因此TF和VEGF之间的双向调控机制促进了肺癌的生长和转移。

### 5.3 TF参与肺癌的局部浸润和转移

TF在各种肿瘤局部浸润和转移中作用已经被广泛地研究。Yu等人发现缺乏TF表达的肿瘤细胞不能在TF低表达的小鼠中生长，却可在正常表达TF的小鼠中生长，说明宿主的TF才是肿瘤生长转移所必需的。实验动物模型^[33-35]^也证明了TF的过度表达或沉默可以促进或抑制肿瘤的恶性生物行为。临床研究中发现肺癌血管侵犯转移者TF的表达明显高于其他肺癌患者^[14]^，采用RNA干扰技术抑制肺癌细胞的组织因子途径抑制物-2(tissue factor pathway inhibitor-2, TFPI-2)，增强了肿瘤细胞的侵袭能力。

肿瘤的浸润转移过程机制复杂，肿瘤细胞首先通过蛋白水解酶如MMP、尿激酶等降解细胞外基质(extracellular matrix, ECM)和基底膜，脱离周边细胞和ECM后借助粘附因子运动，局部浸润或侵入血管和淋巴管扩散到其它部位，最后在新的转移病灶血管形成，肿瘤得以生长。因此这一过程中降解ECM的蛋白水解酶和粘附因子等起到了关键的起始作用。TF通过胞内区和TF-FVIIa复合物激活胞内信号：MMP和尿激酶型纤溶酶原活化受体表达上调，促进ECM溶解酶生成；活化FAK增加肿瘤细胞的粘附性和迁移性。凝血途径下游产生的凝血酶、纤维素和激活的血小板同样促进基质溶解酶MMPs和粘附分子如整合素、P-选择素的产生，参与这一过程。转染正义TF cDNA的肿瘤细胞MMP-2和MMP-9的表达和细胞的侵袭转移能力增加，而转染反义的TF cDNA抑制TF表达则相反降低^[36]^。最新的研究^[37]^在小鼠模型中发现阻断整合素β1或β3可以抑制依赖asTF的主动脉萌芽，asTF结合整合素αVβ3促血管内皮的迁移，因此TF和整合素之间相互作用可能是肿瘤生长转移的机制之一。

## 6 针对TF的肿瘤靶向治疗

肺癌目前已是世界上发病率和死亡率最高的恶性肿瘤之一，除了传统的治疗方法，靶向治疗是现在的研究和应用的热点。通过近二十年来的研究，对于TF在肿瘤发生发展中重要的作用机制认识越来越清楚，因为肿瘤血管内皮细胞和肿瘤细胞异常表达TF而正常的血管内皮细胞不表达，因此针对TF的肿瘤靶向治疗特异性高，对正常组织损伤小，为肿瘤治疗提供了一条新思路。

早期的研究者局限在利用TF的凝血作用来治疗肿瘤。在鼠的实验模型中通过激活肿瘤血管内皮细胞特异性表达TF，使肿瘤血管内形成血栓阻断血流，肿瘤失去供养而坏死，取得了一定的效果^[38, 39]^，但可能导致机体的凝血异常出现DIC或血栓可能游走而使肿瘤转移或正常脏器栓塞限制了其临床应用。后来主要利用TF的桥梁作用，Hu等^[40]^应用突变的FVII(不能启动凝血系统)和Fc结构域组成的免疫交联物，高效的与肿瘤或血管表面的TF特异性结合，通过Fc结构域激活细胞溶解免疫反应，从而杀伤肿瘤。最近Kirkun等^[41]^合成的免疫交联物Icon也是应用相同的原理，但毒性更小。Shoji等^[42]^把合成的EF24 (姜黄素)以FVIIa为载体，在体内与肿瘤的TF结合后被胞吞，释放其细胞毒性作用抑制肿瘤，增强了EF24特异的治疗作用，减少了副作用。随着TF在肿瘤生长中的作用被揭示以及最新siRNA的应用，Amarzguioui等^[43]^利用RNA干扰技术沉默B16黑色素瘤细胞TF的表达可以减少肿瘤在C57BL/6鼠肺部的转移。Fang等^[44]^构建TFsiRNA-pSUPER表达载体，下调人神经母细胞瘤SK-N-MC中TF的表达，从而增强了阿霉素介导的肿瘤细胞凋亡作用。虽然TF靶向治疗的研究在体外或动物实验中取得了进展，但能否应用于临床、药物的安全性和疗效还需长期的研究。
